# Transfer Entropy as a Tool for Hydrodynamic Model Validation

**DOI:** 10.3390/e20010058

**Published:** 2018-01-12

**Authors:** Alicia Sendrowski, Kazi Sadid, Ehab Meselhe, Wayne Wagner, David Mohrig, Paola Passalacqua

**Affiliations:** 1 Department of Civil, Architectural and Environmental Engineering, Center for Water and the Environment, The University of Texas at Austin, Austin, TX 78712, USA; 2 The Water Institute of the Gulf, Baton Rouge, LA 70802, USA; 3 Department of River-Coastal Science and Engineering, Tulane University, New Orleans, LA 70118, USA; 4 Department of Geological Sciences, The University of Texas at Austin, Austin, TX 78712, USA

**Keywords:** transfer entropy, river deltas, model validation, hydrodynamics, process connectivity

## Abstract

The validation of numerical models is an important component of modeling to ensure reliability of model outputs under prescribed conditions. In river deltas, robust validation of models is paramount given that models are used to forecast land change and to track water, solid, and solute transport through the deltaic network. We propose using transfer entropy (TE) to validate model results. TE quantifies the information transferred between variables in terms of strength, timescale, and direction. Using water level data collected in the distributary channels and inter-channel islands of Wax Lake Delta, Louisiana, USA, along with modeled water level data generated for the same locations using Delft3D, we assess how well couplings between external drivers (river discharge, tides, wind) and modeled water levels reproduce the observed data couplings. We perform this operation through time using ten-day windows. Modeled and observed couplings compare well; their differences reflect the spatial parameterization of wind and roughness in the model, which prevents the model from capturing high frequency fluctuations of water level. The model captures couplings better in channels than on islands, suggesting that mechanisms of channel-island connectivity are not fully represented in the model. Overall, TE serves as an additional validation tool to quantify the couplings of the system of interest at multiple spatial and temporal scales.

## 1. Introduction

Numerical simulations of landscapes aim to better understand the dynamics of landscape formation, track their evolution, and make forecasts of their future [1,2,3]. Such models are generally driven by governing laws and rules, and frequently stochastic components, that route mass and energy flows through the environment [1,2]. Land-surface models can generally be broken down into hydrodynamic (i.e., fluid transport) and morphodynamic (i.e., land elevation change caused by erosion and deposition) components, which work in concert to drive landscape evolution [4]. One important application of numerical modeling is to capture hydrodynamic and morphodynamic change in response to changes in initial and boundary conditions (e.g., variable climate, incoming discharge, sediment supply, human impacts) [2]. An important aspect in the development of numerical models, and the focus of this work, is their validation, to ensure that models reproduce the behavior of landscapes accurately (relative to the model’s simplified assumptions) and their results can thus be used to forecast landscapes’ behavior.

Among the features that characterize the Earth’s surface, we focus on river deltas. Coastal deltas are low gradient systems that occur at the interface of land and sea. Millions of people inhabit deltas due to several ecosystem services including productive soils for agriculture and abundant oil and gas supplies [5]. Deltas are influenced by spatially and temporally variable “boundary conditions” that include river discharge, tides, waves, and wind. Interaction of these processes with water, solids, and solutes in the deltaic network lead to spatially complex delta morphologies that display a variety of distributary channel lengths and widths, island sizes, and vegetation patterns [6]. However, the additional impacts of sea level rise (e.g., [7]), subsidence (e.g., [8]), and human-modulated change have led to increased degradation of deltaic systems (e.g., [9,10]). Coastal Louisiana, for example, has seen over 3900 km^2^ of land loss over the last 150 years (e.g., [11,12,13,14]). These impacts have motivated research into engineered solutions to mitigate land loss and in the development of numerical models to project deltaic land change.

One type of restoration intervention, currently applied in coastal Louisiana [15], is controlled river diversions that route sediment and water through artificial breaches in river levees with the aim of building land through natural processes (e.g., [16]). Diversions have been analyzed for their land building potential, their sediment and nutrient dynamics, and their influence on the adjacent river morphology [17,18,19,20,21,22,23]. This research on coastal restoration adds to a body of work on river delta formation (e.g., [4,24,25,26]), mouth bar evolution [27,28,29]), and fluvio-delta relationships (e.g., [30,31,32]), often studied with numerical modeling and physical experiments. The processes of delta evolution occur over thousands of years and are highly dynamic, and implemented diversions take time to develop landforms. Thus, numerical modeling can assist in decision making by providing a better understanding of land building under given conditions and generating predictions of future landforms. A main question then arises: given the importance of models in the decision making process of coastal restoration and in the understanding of deltas, how can we robustly validate these numerical models?

Hydrodynamic model validation generally occurs through comparison of modeled and field observations or comparison with theoretical solutions of the flow behavior under given conditions. Comparison of time series (e.g., whether the model reproduces observations of river discharge or stage) is accomplished through statistics such as the Nash-Sutcliffe efficiency [33], the coefficient of determination, and the root mean square error (RMSE) among others [34,35]. Many of these model-field comparison metrics are applied to time series at single locations only, thus spatial information that is relevant to system dynamics is not accounted for during model validation. Comparison with theoretical solutions (e.g., whether the model reproduces the predicted gradually varied flow profile) allows for spatial relationships to be validated from modeled outputs. For example, Liang et al. [4] simulated flow over a bump and flow partitioning at a bifurcation using a hydrodynamic model, and validated those results by comparing the spatial distribution of flow and the local flow hydraulics generated by the model to established results from other models and theoretical solutions. Additional metrics proposed for deltaic systems aim to better link delta morphology with the processes controlling morphology. Edmonds et al. [36] investigated a number of delta metrics to compare results among field, experimental, and modeled deltas. Passalacqua et al. [37] introduced a statistical framework for analyzing delta networks, and quantified several measures in the Ganges-Brahamputra-Jamuna Delta. While the hydrodynamic and morphodynamic validation techniques described above are quantitative, they generally only measure relationships across space or time, and they cannot be used to compare across multiple temporal or spatial scales. These validation tools are also unable to measure important relationships between different system processes (e.g., river discharge, tides, water level), called couplings, that characterize a system. With the aim of both quantifying these couplings, and assessing whether the model can reproduce these couplings, we explore the statistics of information theory.

Information theory (IT) [38] is a branch of mathematics that uses information from the probability density functions of random variables to measure the communication of information among variables, and quantifies the uncertainty inherent in a random variable. We propose that the tools of IT provide a means to robustly validate numerical modeling results. By using information from the pdf, there is no need for assumptions on the linearity of relationships or on the prior distribution of variables. Tools such as mutual information (MI) and transfer entropy (TE) [39] quantify the information shared and transferred among variables. MI measures the synchronization among variables, similar to correlation statistics, yet MI can be applied for multi-dimensional variables that have nonlinear dependencies. TE captures the conditional reduction in uncertainty of a target variable due to knowledge of another variable, thus directional relationships can be investigated, where directionality is expressed as the influence of one variable on the other. TE is thus a more powerful tool over the traditional correlation coefficient due to its directionality and its ability to accurately measure nonlinear relationships. Leopold and Langbein [40] discussed the use of IT statistics to predict the evolution of the river profile. In deltas, Tejedor et al. [41] proposed using IT as a means to quantify the topological complexity of deltaic systems, while Sendrowski and Passalacqua [42] used IT to quantify couplings of deltaic water levels, discharge, tides, and wind. IT has also been employed to quantify dynamical interactions in climate systems (e.g., [43,44]) in ecohydrological applications, [45,46], and for hydrological models (e.g., [47,48,49]).

We propose using TE as a validation tool for hydrodynamic models. TE measures information transfer from one variable to another, thus relationships among external drivers and modeled hydrodynamics (model couplings) can be compared to observed data relationships (observed couplings). Furthermore, TE quantifies directionality among interacting time series, allowing for a more rigorous analysis of system dynamics, and has advantages over MI, a directionless measure. Different system states can be identified using TE, where “states” can refer to various time periods, spatial scales or signal frequencies, providing a means of exploring spatial and temporal dynamics. Since TE quantifies directional information transfer, several works have discussed the relationship between TE and causality [50,51,52]. While distinctly different, TE is measured when causal interactions occur [51], therefore more meaningful interpretations can be made of the measured deltaic dynamics using TE as a validation tool and analyzing couplings in the system compared to traditional correlation measures.

Viewing the deltaic network as a collection of drivers (e.g., discharge, wind, tides) and sinks (e.g., water, sediment) that exchange information with each other (termed process connectivity, [42,45,53,54,55]) we use TE to quantify information transfer from river discharge, tides, and wind to water level measured at several locations in a river delta. Sendrowski and Passalacqua [42] used this approach using data collected at the Wax Lake Delta (LA, USA) to quantify the timescale and strength of wind, tides, and discharge on water levels at five islands and one channel. Here, we compare their field results to modeled water levels along with additional field and modeled measurements from three channel locations. In the following work, we discuss the field site, modeling approach, and application of information theory (Section 2). We then compare observed and modeled hydrodynamic data for islands and channels in Wax Lake Delta (Section 3) and discuss how well the model reproduces deltaic couplings, the use of TE as a tool for hydrodynamic model validation, and its application to other modeling efforts (Section 4). We also discuss the limitations of this study (Section 4), and present the conclusions of this work (Section 5).

## 2. Materials and Methods

### 2.1. Study Site and Field Data Collection

**Figure 1:**
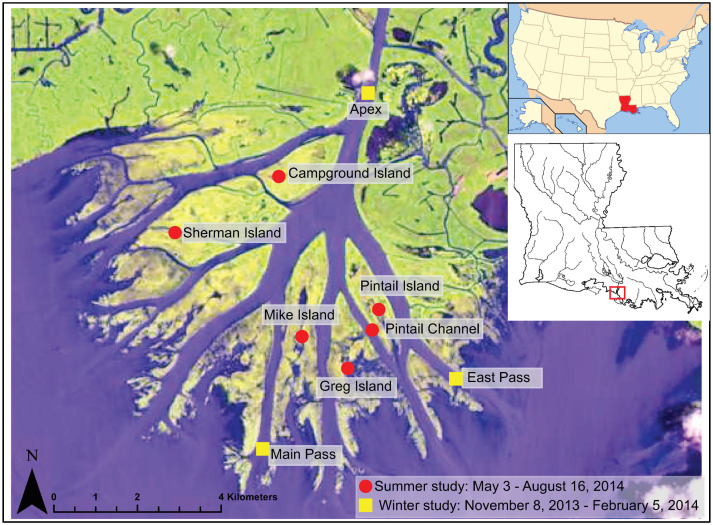
Map of Wax Lake Delta (WLD) showing the locations of the field and modeling analysis. The circles are from the summer field campaign measuring water level on five islands and one channel from 3 May to 16 August 2014. The yellow squares are channel data from a field campaign from 8 November 2013 to 5 February 2014. Image is a Landsat 8 satellite photo from 19 June 2014, courtesy of the United States Geological Survey (USGS). Inset images show location of Louisiana in the USA and the location of WLD in Louisiana.

Wax Lake Delta (WLD) (Figure 1), the study area of interest, is a 150 km^2^ delta located in coastal Louisiana, USA. WLD emerged after an engineered diversion was constructed in the 1940’s to prevent flooding [56,57], thus, WLD is often considered an analogue of river diversion projects. An area of little human interference and relatively small extent, WLD has been the site for extensive field, remote sensing, and modeling efforts, such as those exploring the dynamics of channels [58,59], islands [60,61], vegetation [62,63], and shoreline and deltaic evolution [57,64,65].

WLD displays high surface water connectivity, with up to 54% of flow entering islands from the distributary channels [60,66]. Islands are characterized as areas of high biogeochemical processing and are bordered by levees that become increasingly subaqeous downstream and are interspersed with secondary channels [60]. Large vegetation patches exist on the islands, whose extent is controlled by the hydroperiod. Patches generally emerge in the summer and senesce in the winter [67].

Despite being considered a river dominated delta, WLD has a 40 cm tidal range which is able to significantly affect water level on the delta [42,64]. The median discharge entering WLD is 3000 m^3^ s^−1^, about 30% of the Atchafalaya River flow, which is WLD’s feeder channel. Typical of the Louisiana coast, WLD experiences cold fronts and South wind events that can exert strong control on water and sediment transport in and out of the delta (e.g., [68,69]). South wind events cause water level set-ups on islands that lead to increased surface water connectivity among locations, thus wind acts as a driver of process connectivity. [42]. Outside of wind and tidal events, water levels across the delta show strong synchronization with each other, dominated by tides over daily timescales, and discharge over longer timescales [42]. This process connectivity is dependent on the strength of drivers and vegetation density [42].

To better quantify island and channel hydrodynamics, two sets of water level data were collected, one in channels and one on islands. The island dataset is discussed in [42] and is briefly summarized here. Six Solinst Water Levelogger Junior Edges were installed on five islands and in one channel from 5 May to 16 August 2014 (the locations of the measurements are shown as circles in Figure 1). The instruments have an accuracy of ± 10mm and took measurements every 6 minutes. Water levels in the channels were collected from 8 November 2013 to 5 February 2014. Three Current Temperature Depth loggers (CTDs) were installed at the apex of the delta and at the proximal ends of East and Main passes (squares in Figure 1). The instruments took measurements of water level every minute. Locations for both the island and channel water level measurements were chosen to cover a large and diverse portion of the delta. Observations of river discharge, tides, and wind (external drivers of water, solid, and solute transport at WLD) were collected from the USGS and the National Oceanic and Atmospheric Administration (NOAA), respectively. River discharge measurements were collected from the USGS Calumet gauge (Gauge #07381590) located 17 km upstream of WLD. Tides, wind speed, and wind direction data were collected from the NOAA tides and Currents Amerada Pass station (Station #8764227) located 10 km east of WLD in the Atchafalaya Delta. The discharge data were collected at 15 min intervals and the tide and wind data at 6 min intervals.

### 2.2. Model Set-Up

Model simulations were performed using Delft3D, a process-based three-dimensional numerical model developed by Deltares in the Netherlands. Delft3D has integrated modules to simulate hydrodynamic, sediment transport and morphological changes [70]. The numerical scheme of the model is based on finite differences. The model solves the unsteady shallow-water equations in a rectangular or curvilinear or spherical grid; the grid is assumed to be orthogonal and structured. The system of equations consists of the momentum equation, the continuity equation, the transport equation, and a turbulence closure model. The hydrodynamic module calculates water movement by solving the system of equations and produces temporal and spatial outputs of hydrodynamic variables such as water depths, velocity, etc. The model simulations for this study were done in two-dimensional depth averaged mode, as is typically done in these systems due to the two-dimensional nature of the flow. Delft3D is a well established model for studying delta dynamics, and has been used in previous studies to measure delta change for various initial and boundary conditions (e.g., [30]).

The model domain includes the Wax Lake Outlet (WLO) channel, the entire WLD complex and a portion of the Gulf of Mexico (Figure 1). The modeling grid has a resolution of 45 × 60 m and 100 × 300 m, with higher resolution at the delta complex. The model bathymetry was derived primarily from [71], combined with Light Detection and Ranging (LiDAR) survey of the overbank areas and the floodplains on each side of the WLO channel. The model has two open boundaries: the upstream boundary for water inflow and the tidal boundary at the downstream end. The hydrodynamic boundary conditions are the river discharge from the USGS Calumet Gauge, while wind and tides are measured at the NOAA Lawma-Amerada Pass station. The model propagates wind across space using hourly wind from the NOAA Lawma-Amerada Pass station. The external drivers acting on field and model data are the same, providing a more robust comparison between field and modeled couplings.

The hydrodynamic calibration was performed using water level measurements collected in WLD [42,60]. The bed roughness was the primary parameter used in the hydrodynamic calibration process. Bed roughness was parameterized with Chezy values adjusted until the simulated and measured water levels had a correlation coefficent of at least 0.88 and an RMSE less than 0.2 [72]. Finally, roughness values of 55 for islands and 100 for channels were selected.

The model generated water level measurements at 15 min intervals at all locations shown in Figure 1 for the two time periods that field data were collected.

### 2.3. Information Theory

Uncertainty in a random variable, or signal, is quantified using the Shannon entropy (*H*), given by the expression:

(1) $$H\left(X\right)=-\sum_{i=1}^{N}p\left(x_{i}\right)log_{2}\left[p\left(x_{i}\right)\right]$$

where *N* is the number of outcomes of the random variable *X* and $p(x_{i})$ is the probability of each outcome. For example, a variable with a uniform distribution has maximum uncertainty, and thus maximum entropy *H*, given that each outcome has equal probability of occurring. *H* and all subsequent IT metrics are measured in bits of information.

Information flow occurs when random variables interact with each other. If random variable *X* informs on the values of another random variable, *Y*, (through sharing or transferring of information) a reduction in uncertainty occurs in random variable *Y*.

Two measures used to quantify signal interaction are mutual information (MI) and transfer entropy (TE). MI measures the amount of shared information between two variables:

(2) $$MI\left(X_{\tau },Y\right)=\sum_{x_{t-\tau },y_{t}}p\left(x_{t-\tau },y_{t}\right)log\frac{p(x_{t-\tau },y_{t})}{p\left(x_{t-\tau }\right)p\left(y_{t}\right)}\;$$

We show the time-lagged MI in Equation (2), where τ is the time lag and $p(x_{t-\tau },y_{t})$ is the joint pdf of signals *X* at time *t*-τ and *Y* at time *t*. MI also measures the dependency or synchronization between two variables and can quantify asynchronous processes [73].

TE measures the amount of information transfer from one variable to another [39]:

(3) $$TE\left(X\to Y\right)=\sum_{y_{t},y_{t-\Delta t},x_{t-\tau \Delta t}}p\left(y_{t},y_{t-\Delta t},x_{t-\tau \Delta t}\right)log\frac{p(y_{t}|y_{t-\Delta t},x_{t-\tau \Delta t})}{p(y_{t}|y_{t-\Delta t})}\;$$

where τ is the time lag, t−Δt represents the history of the variable, $p(y_{t}|y_{t-\Delta t})$ is the probability of random variable *Y* conditioned on its own past, and $p(y_{t}|y_{t-\Delta t},x_{t-\tau \Delta t})$ is the probability of *Y* conditioned on the past of *Y* and the time-lagged past of *X*. Stating TE in terms of the Shannon entropy:

(4) $$TE\left(X\to Y\right)=H\left(y_{t}|y_{t-\Delta t}\right)-H\left(y_{t}|y_{t-\Delta t},x_{t-\tau \Delta t}\right)$$

Thus, TE measures the difference between the reduction in the uncertainty of *Y* due to knowledge of *Y*’s own past and the reduction in uncertainty of *Y* due to knowledge of the past history of *Y* and the time-lagged history of *X*. This difference increases as *X* or *X* and *Y* together provides more information to *Y* than previous values of Y alone. TE is a directional measure, in that *X* may provide information to *Y*, but *Y* may not inform *X*. The time lag identifies the characteristic timescale of information flow, which occurs at the onset of statistically significant information transfer.

### 2.4. Data Processing and Process Networks

In order to calculate TE relationships, all variables within a dataset must be measured at the same timestep. The three data sets analyzed (observed island water levels, observed channel water levels, and modeled water levels on islands and channels) were collected at different resolutions (6, 1, and 15 min intervals, respectively). All datasets were subsampled to some degree, given that tide and wind data were collected at 6 min intervals and discharge data were collected at 15 min intervals. The observed island and channel water level data were subsampled to include as many observations of the original data as possible without extensive upsampling of the discharge data. Therefore, the observed island water level data were analyzed at 12 min intervals (with wind, tide, and discharge data sampled at 12 min as well), while the observed channel data were subsampled at 10 min intervals (along with tide, wind, and discharge data for that time period). The modeled data was kept at 15 min and the tide and wind data for those periods were subsampled to 15 min. Thus, the three datasets (observed island water levels, observed channel water levels, and modeled island and channel water levels) were analyzed at 12, 10, and 15 min, respectively.

Since information flow can occur at multiple scales, we filter the data to isolate timescales of interest. Following the approach of Sendrowski and Passalacqua [42], the water levels for all three datasets are filtered twice: once to remove the tidal fluctuations from water level and again to remove the long-term fluctuations, thereby preserving data with periods of <10 h (referred to as high frequency water level fluctuations). The discharge data are filtered to remove the high-frequency and tidal fluctuations, preserving periodicities >1.5 days. The wind speed and wind direction data are combined by multiplying negative wind speed and the cosine of wind direction, referred to in the rest of this work as -WCosA or wind. This wind signal is filtered similarly to discharge to emphasize longer-term wind events (occuring over 1–5 days) rather than short gusts. All filtering is performed using a fifth order Butterworth filter [74].

To capture different states of the system through time, we partition all time series into ten-day windows. Ten days encompasses wind events and spring and neap tidal transitions in this deltaic system. Each window is shifted by one day, for a total of 96 windows covering May–August 2014 for the island dataset and 81 windows covering November 2013–February 2014 for the channel measurements, for field and modeled data. A ten-day window also ensures there is enough data to create a representative pdf [45].

For each dataset, TE is quantified for every possible coupling in each window over 48 h of lag, using the software developed by Ruddell [75]. While all couplings are quantified, in this work we only analyze the directional coupling of external driver → water level, to compare field and model relationships. We do consider both directions when analyzing couplings of water level ↔ water level. All pdfs are created with 11 bins using a local binning scheme. The statistical significance of the TE results are found using bootstrapping methods, as discussed in Ruddell and Kumar [45].

We visualize results using process networks; variables serve as network nodes and statistically significant TE relationships are network links [42,45,55,76]. By viewing the process network through time, we see how individual links change, and get a sense of the spatial variation of relationships across the delta. Links are associated with a directionality, timescale, and strength, each quantified using TE. The sum of the links in the networks is the number of couplings, and we also view this number through time, to understand bulk system behavior. Our validation is through four means: (i) by observing the process networks, to see how the model captures relationships across space within a window, (ii) by measuring the number of couplings through time, to measure how well the modeled data captures longer-term change, (iii) by plotting TE for individual relationships, and (iv) by viewing the characteristic timescale of information flow for all considered couplings, to see how well the model captures the couplings in strength and timescale.

## 3. Results

### 3.1. Comparison of Island Hydrodynamics Based on Observed and Modeled Data

**Figure 2:**
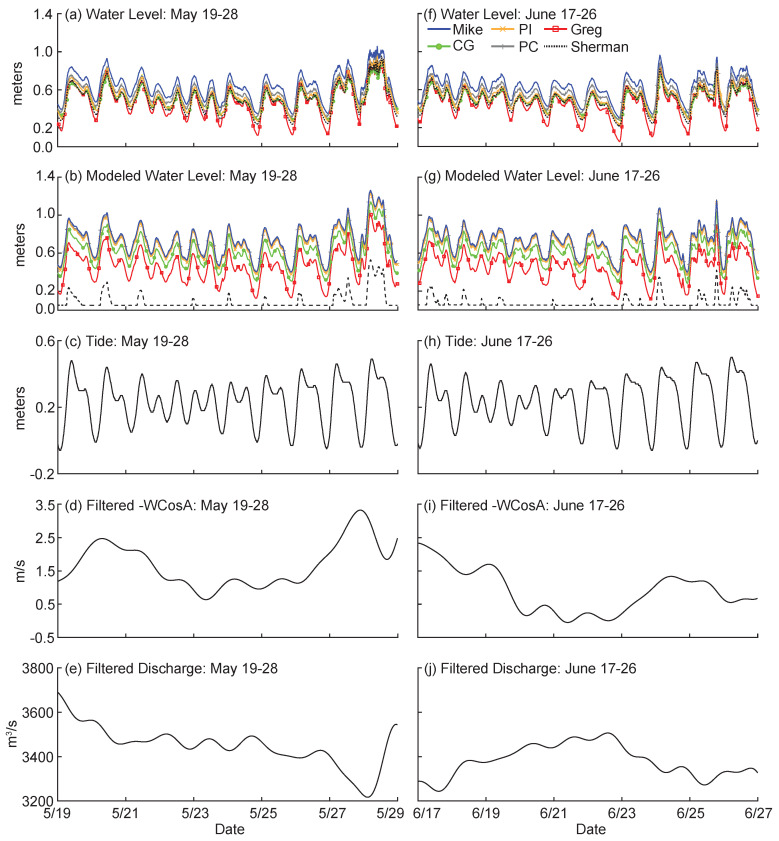
Time series of water level, modeled water level, tide, filtered wind, and filtered discharge for two time periods (**a**–**e**) 19–28 May and (**f**–**j**) 17–26 June 2014. For the water levels, PI = Pintail Island, PC = Pintail Channel, and CG = Campground Island. Wind and discharge are filtered using a 5th order Butterworth filter.

We compare the observed and modeled time series and their corresponding TE results over two periods: 19–28 May and 17–26 June 2014. These windows have been chosen based on high process connectivity measured by Sendrowski and Passalacqua [42]. For the 19–28 May window, field and modeled water levels show daily tidal fluctuations (Figure 2a,b). Modeled water level on Sherman Island shows no water for most of the window, with fluctuations only occurring around some high tides (dashed line in Figure 2b). Around 28 May, field water levels show high frequency fluctuations over several hours that are not present in the modeled water level data (Figure 2a,b). Over the window, the tide transitions from neap to spring (Figure 2c) and the filtered wind time series shows the presence of mostly South winds, peaking at a speed of 3.5 m/s on 28 May (Figure 2d). Filtered discharge is decreasing during this time, from 3700 m^3^ s^−1^ to 3200 m^3^ s^−1^ (Figure 2e).

In the 17–26 June window, modeled and field water levels show daily tidal fluctuations (Figure 2f,g), though modeled water level at Sherman Island shows little fluctuation. Tide transitions from neap to spring (Figure 2h), filtered wind decreases from a South wind to a weaker mixed North/South wind (Figure 2i) and filtered discharge remains between 3600 and 3200 m^3^ s^−1^ (Figure 2j).

**Figure 3:**
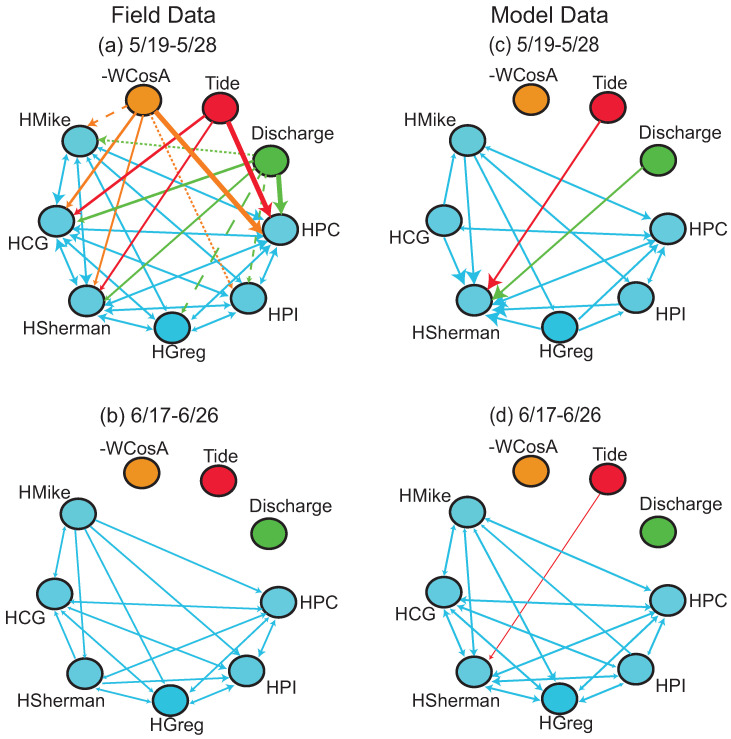
Process networks depicting statistically significant TE from tides, discharge, and wind (-WCosA) to the high frequency water level fluctuations (HLocation) and among high frequency water level fluctuations for the (**a**,**b**) field and (**c**,**d**) modeled island data for two time periods: 19–28 May and 17–26 June 2014. Within a network, arrows from tide, discharge, and wind are arranged in order of most persistent (thickest line) to least persistent (dashed line) for each location. The color of the arrow identifies the driver node. Among water levels, the biggest arrowheads refer to the most persistent relationships. For the water levels, CG = Campground Island, PI = Pintail Island, PC = Pintail Channel.

Process networks for the 19–28 May and 17–26 June windows show differences in couplings between the field and modeled data (Figure 3). The process network for the field data for 19–28 May shows high connectivity, with connections from drivers to high frequency water level fluctuations at all locations and connections among island locations as well (Figure 3a). Information transfer from external drivers and other locations in WLD is most persistent (i.e., has the most number of statistically significant time lags) at Pintail Channel (PC) and Campground Island (CG). Most driver connections are not captured in the modeled data process network for 19–28 May (Figure 3c). Information transfer is measured only from tide and filtered discharge to the high frequency water level fluctuation on Sherman Island. Among water levels, Sherman Island is most influenced, followed by Pintail Channel. Many of the connections among water levels are also not captured, such as those towards Campground and Mike Islands.

No driver-water level connections are measured in the 17–26 June window, however, connections among observed high frequency water level fluctuations are captured and are most persistent at Pintail Island and Pintail Channel (Figure 3b). More links are captured in the modeled data process network for 17–26 June than in the field one (Figure 3d). For example, tide transfers statistically significant information to Sherman Island high frequency water level in the modeled data, and more connections are seen among different locations (such as Campground → Sherman).

**Figure 4:**
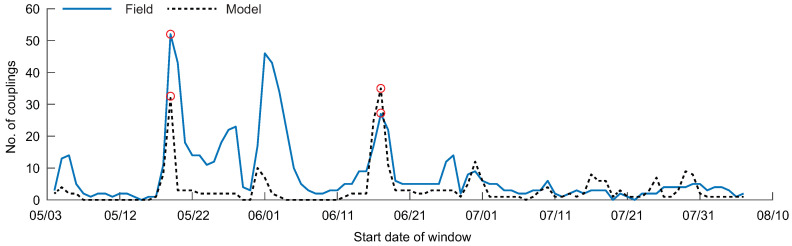
The number of statistically significant transfer entropy (TE) couplings for all windows investigated in the study, for the field (solid line) and modeled (dashed line) data. The circles show the window for the process networks depicted in Figure 3.

Summing the number of links in each process network and viewing this number for all windows, we see that couplings from modeled results are overall underestimated compared to results from the field data (Figure 4). However, there are windows where the modeled number of couplings is overestimated, such as in the 17–26 June window. Both field and modeled data show a decrease in the number of couplings through time, starting with windows in mid-June.

**Figure 5:**
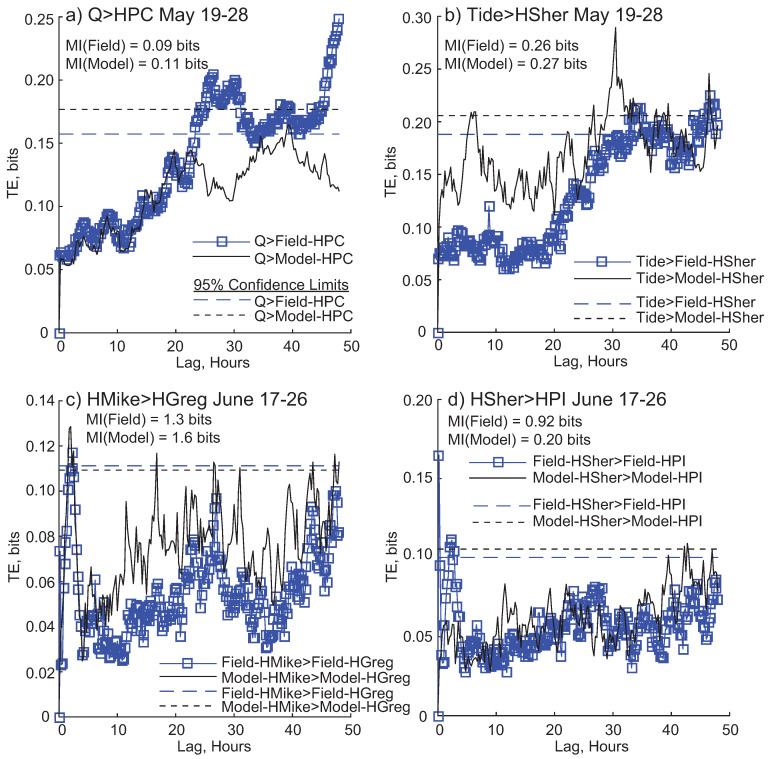
Transfer entropy (TE) results within a window. Dashed lines are 95% confidence limits. (**a**) Information transfer from filtered discharge (Q) to high frequency modeled and field water level for Pintail Channel (HPC) for the window 19–28 May; (**b**) TE from tide to Sherman Island high frequency water level (HSher), for field and model data, for the 19–28 May window; (**c**) TE from high frequency water level on Mike (HMike) to high frequency water level on Greg (HGreg) from 17–26 June; (**d**) TE from high frequency water level fluctuations on Sherman Island (HSher) to high frequency water level fluctuations on Pintail Island (HPI) from 17–26 June. Each window also shows the mutual information (MI) value for that relationship.

Next we compare TE results within a window over multiple time lags, to see the similarity of the TE links among field and modeled data. Over 19–28 May, filtered discharge (Q) provides statistically significant information to the field collected high frequency water level fluctuations at Pintail Channel (PC) (Figure 5a). Information transfer begins around 25 h of lag. The discharge → Pintail Channel relationship for the modeled data never becomes statistically significant. Tide transfers significant information to field and modeled water level at Sherman Island, though differently; TE becomes significant at an earlier time lag for modeled Sherman than field Sherman (Figure 5b). In the 17–26 June window, modeled (and field) high frequency water level at Mike Island transfers significant information to Greg Island (Figure 5c). TE results between field and model are different for the Sherman → Pintail Island (PI) relationship; field collected water level at Sherman transfers information to PI much earlier than the modeled coupling (Figure 5d).

The characteristic timescale of information transfer (the time lag at which information transfer begins) is collected for all relationships analyzed in every window to obtain the range and average information transfer for field and modeled couplings (Table 1 and Table 2). The average timescales of information transfer from drivers to Sherman Island water level compare well between field and modeled data, despite Sherman Island often being out of the water, though the ranges tend to be wider for modeled couplings for tide and wind relationships. No information transfer is measured for any window from drivers to Mike, Campground, Greg, Pintail Island, and Pintail Channel water level in the modeled data. The average timescale of tidal information transfer to field water levels occurs around 25 h of lag for all locations, while the average wind timescale is highly variable for all locations.

Among water levels, timescales of information transfer are highly variable for field and modeled data. The average timescale of information transfer from Mike, Campground, and Sherman Island water levels to the six water levels is higher in the model data compared to the field data. Many information transfer relationships from Greg, Pintail Island, and Pintail Channel water levels to the six water levels have lower average timescales in the modeled data than the field data.

### 3.2. Comparison of Channel Hydrodynamics Based on Observed and Modeled Data

**Figure 6:**
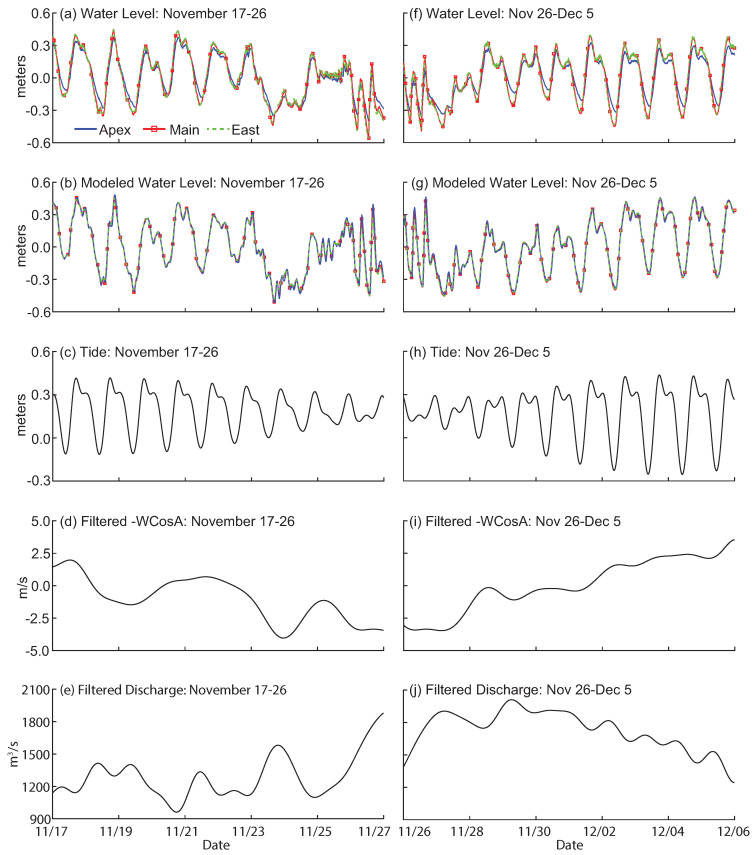
Time series of water level, modeled water level, tide, filtered wind, and filtered discharge for two time periods (**a**–**e**) 17–26 November and (**f**–**j**) 26 November–5 December 2013. Wind and discharge are filtered using a 5th order Butterworth filter.

Detrended water levels for the field and modeled data compare well for the window 17–26 November (Figure 6a,b). All water levels show daily tidal fluctuations, with a deviation from the tide around 24 November. In this window, the tide transitions from spring to neap (Figure 6c) and winds are mostly from the North (Figure 6d). Discharge increases from 1200 m^3^ s^−1^ to 1800 m^3^ s^−1^ (Figure 6e).

Daily tidal fluctuations are measured for field and modeled water level for the 26 November–5 December time period, though the fluctuations for modeled water level at Apex are stronger than the field measured water level at the same location (Figure 6f,g). Tide transitions from neap to spring (Figure 6h) and wind transitions from a North wind to a South wind (Figure 6i). Discharge decreases from 1900 to 1200 m^3^ s^−1^ (Figure 6j).

**Figure 7:**
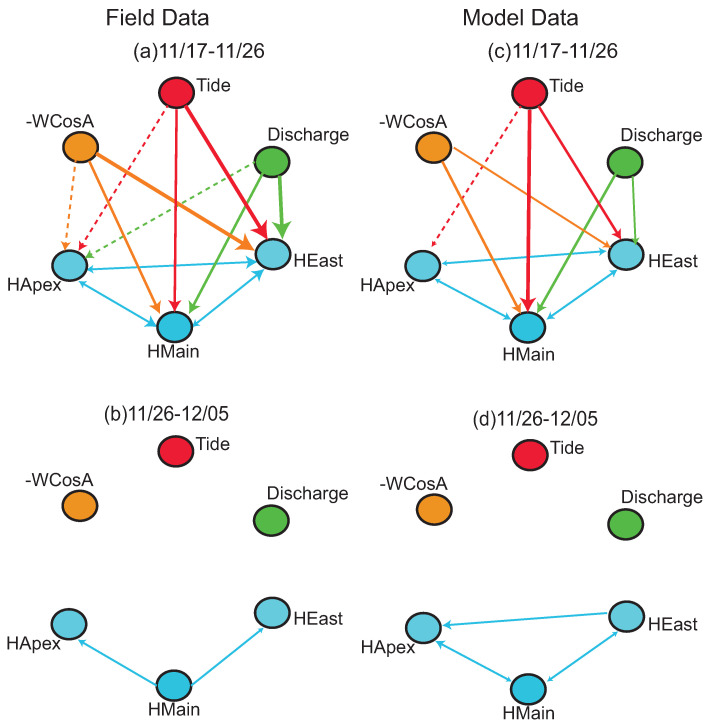
Process networks for high frequency water levels (HLocation) showing the (**a**,**b**) field and (**c**,**d**) modeled process connections for two time periods: 17–26 November and 26 November–5 December 2013. Within a network, arrows from wind (-WCosA), tide, and discharge are arranged in order of most persistent (thickest line) to least persistent (dashed line) for each location. The color of the arrow identifies the driver node. Among water levels, the biggest arrowheads refer to the most persistent relationships.

Model- and field-based process networks compare well for the 17–26 November window (Figure 7). For the field data, all locations experience statistically significant TE from the three external drivers; the most persistent relationships for the high frequency water level are at East Pass, followed by Main Pass and Apex (Figure 7a). In the modeled process network for the same period, the Wind → Apex and Discharge → Apex links are not captured and the tide relationship is now more persistent at Main Pass than East Pass (Figure 7c).

Most couplings become insignificant in the 26 November–5 December window for the field and modeled data (Figure 7b,d). The modeled data measures more process connections among water levels than the field data; extra links are measured for Apex → Main, East → Main, and East → Apex high frequency water levels.

**Figure 8:**
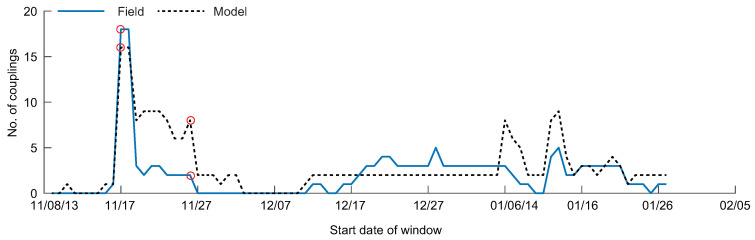
The number of statistically significant transfer entropy (TE) couplings for all windows investigated in the study for the field (solid line) and modeled (dashed line) data. The circles show the window for the process networks depicted in Figure 7.

Viewing the number of couplings through time, the modeled data overestimates the number of couplings for many windows (Figure 8). However, the overall trend in the field data, with peaks in connectivity around the 17 November windows followed by a small number of connections through December and January, is also captured by the modeled data.

**Figure 9:**
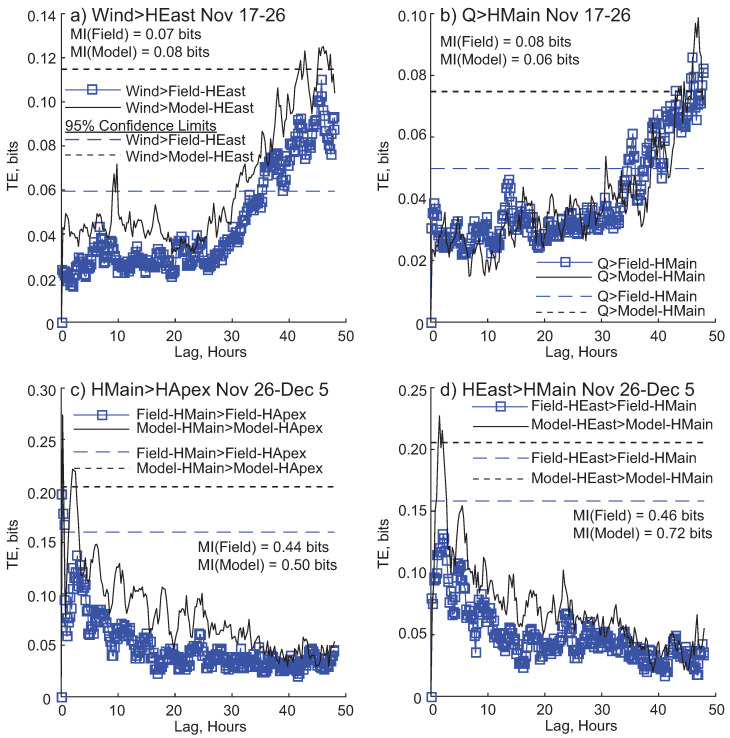
Transfer entropy (TE) results within a window. Dashed lines are 95% confidence limits. (**a**) Information transfer from filtered wind (FWCosA) to high frequency modeled and field water level for East Pass (HEast) for the window 17–26 November; (**b**) TE from filtered discharge (Q) to Main Pass high frequency water level (HMain), for field and modeled data, for the 17–26 November window; (**c**) TE from high frequency water level on Main (HMain) to high frequency water level at Apex (HApex) from 26 November–December 5; (**d**) TE from high frequency water level fluctuations at East Pass (HEast) to high frequency water level fluctuations at Main Pass (HMain) from 26 November–5 December. Each window also shows the mutual information (MI) value for that relationship.

TE results within a window show differences between field and modeled data (Figure 9). The wind → East Pass high frequency water level relationship for the modeled data displays the same shape as the field-based relationship in the 17–26 November window, though significant information transfer occurs later and is less persistent (Figure 9a). Filtered discharge (Q) on modeled Main Pass high frequency water level is stronger than the relationship with field collected Main Pass, however the link becomes significant at a later lag (Figure 9b). In the 26 November–5 December window, connections among water levels are very similar between field and model; the Main → Apex link shows similar strength and timescale of information flow (Figure 9c). However, though the shape of the East → Main TE relationship is similar for field and modeled data, the link is only significant for the modeled data (Figure 9d). For all relationships shown, the threshold level for statistical significance of the model relationships is higher than the field relationships.

The characteristic timescale of information transfer from drivers to the high frequency water level fluctuations in channels is higher for East Pass in the modeled data, while it is lower for all Main Pass water level relationships except Tide → Main Pass (Table 3 and Table 4). The modeled data has a higher average information transfer timescale for Tide → Apex water level, while the Wind → Apex water level timescale is very similar to the field timescale for that relationship.

Among water levels, the average timescales of information transfer are overall much higher in the modeled data than the field data, and the ranges are also wider . The modeled data does not capture the transfer of information from Main and East water levels to Apex water level.

## 4. Discussion

### 4.1. Comparison of Observed and Modeled TE Couplings

We compared TE results within a window for different spatial locations, through time, for individual relationships over a range of time lags, and at the characteristic timescale of information flow. For all results, information transfer from drivers to observed and modeled water levels occurs in the high frequency fluctuations of water level. For the observed island data, the 19–28 May window is a period of high connectivity due to a South wind event around 28 May that causes water level set-ups (Figure 2a, discussed further in Sendrowski and Passalacqua [42]). The modeled data in this time period also exhibits water level set-ups (Figure 2b), however, the high frequency fluctuations caused by the wind event are not captured and therefore no statistically significant TE links are quantified from any external driver in the process network (Figure 3c). Given that the model propagates wind across space using hourly wind, rather than a space varying wind at a fine resolution, water level set-ups occur during wind events, but without the high frequency fluctuations that are present in the observed water level. However, couplings among modeled water levels increase during these wind events, as all locations are experiencing the wind event and transfer that information to each other. The strength and timescale of those water level relationships are highly variable, given that each location is influenced by wind, tides, and discharge differently.

Observing the number of couplings through time, three windows show high connectivity for the field data: 19–28 May, 1–10 June, and 17–26 June (Figure 4). Each of these windows corresponds to a South wind event, where driver-water level TE relationships were detected (except for the 17–26 June window where we hypothesize the wind event occurred prior to sensor measurement at the Amerada Pass Station, due to the prevailing wind direction, discussed further in [42]). The connections measured with the model data underestimate the connectivity for most windows, likely due to missing information from the high frequency fluctuations of water level. Some windows show overestimated couplings from the model data, beginning in mid-June. During this time in WLD, incoming discharge is decreasing, wind is weakening from South wind to mixed North/South wind, and vegetation cover is increasing on the island tops [42,61]. The lack of vegetation in the model may lead to smoother paths between water levels at different locations, resulting in overestimated TE relationships. However, the bulk behavior of the system is captured through time.

The model does capture driver connections to Sherman Island high frequency water level. The time series of modeled water level shows that Sherman has little water much of the time, with fluctuations occurring during rising tides. The 45 × 60 m grid size on the islands misses smaller scale features, such as small secondary channels, that deliver water directly to the islands. This lack of small features results in island locations, such as Sherman Island, with less surface water connectivity to the adjacent channels, and therefore differences in TE relationships, compared to the other island locations. Modeled Sherman appears to be primarily tidally driven, and accordingly statistically significant tide → Sherman TE is quantified in the May 19–28 and 17–26 June windows. Within the 19–28 May window, the TE of tide → modeled Sherman is similar in shape to the field relationship, but is overall stronger. Sherman Island is also the location of highest connectivity in the two windows, likely due to the tidal signature present at the other locations, which transfer that information to the Sherman Island water level.

The shape of the TE relationships within the window looks relatively similar for all couplings shown, despite several lags where statistically significant information transfer is not occurring. Given that the relationships should be the same, a comparison of the shape of the TE relationship provides some insight into how the model propagates information over a time lag. For example, the discharge → Main Pass high frequency water level relationship (Figure 9b) displays fluctuations in the TE for modeled data over the 48 h of lag, before TE becomes significant around 45 h of lag. The field-based relationship also shows fluctuations however these are smaller and the coupling becomes significant much earlier. For many couplings, the modeled data must overcome a higher threshold than the field data to be considered statistically significant. Given that the model has less variability than the field (as seen in the lack of high frequency fluctuations of water in the modeled data), a higher significance threshold must be surpassed to observe information transfer between processes. Many modeled couplings also have higher zero-lag MI than the field couplings, indicating that the modeled results are more synchronized, likely due to the lower varibility of modeled data compared to field observations.

While water levels on the islands respond strongly to South wind events, water levels in the channels also respond to North wind events. In the 17–26 November window, a North wind event around 24 November causes water levels to deviate from the diurnal and semidiurnal tidal fluctuations (Figure 6a). Connections are measured from drivers to the three channel locations for the observed and modeled data (Figure 7). Connections among water levels are measured for the observed and modeled data in the 26 November–5 December window, as water levels become increasingly tidal (Figure 6f,g). The shape of the TE plots reveal that connections are very similar for field and modeled data, for driver interactions and for relationships among water levels for the two time periods (Figure 9). For example, the field and modeled coupling of wind → East Pass water level and discharge → Main Pass water level show similar shapes, significant timescales, and strengths. This similarity indicates that field and model boundary conditions transfer information very similarly. Connections among water levels also look very similar between field and modeled data, indicating that spatial connectivity is captured by the model.

Overall, the model appears to capture channel couplings better than island connections. This behavior is to be expected, despite the model calibration with the island water levels, since channels are more connected to the river and tidal basin than island locations, which have more complex morphologies and tend to be rougher. Capturing channel-island connectivity can be challenging for models, as seen for the modeled Sherman Island water level, and this connectivity has only recently been explored in field and numerical modeling [60,66]. Also, all channels (Chezy roughness value 100) and islands (Chezy roughness value 55) are parameterized using spatially homogeneous roughness values. Channels in WLD have been shown to have variable bed cover [58], while islands display spatial heterogeneity in vegetation patch size, species, and density, and temporal changes due to vegetation seasonality. The homogeneous roughness values in the model have the effect of “smoothing” the path between locations, which likely leads to an increase in information transfer among water level at different locations. This difference may explain the overestimation of couplings by the modeled data for the islands at the end of the summer, and for many windows in the channel data. Despite this spatial homogeneity, the model provides a good estimate of connectivity across the system, both in how drivers affect different locations and in the behavior of those interactions through time.

### 4.2. TE as a Model Validation Tool

Directional couplings from external drivers to deltaic water levels have only recently been quantified using TE [42] and these couplings can now be quantified in modeled data as well. While other hydrodynamic validation methods, such as the RMSE and r^2^ statistic, compare observed and modeled data directly, TE serves as an additional level of validation by quantifying how well the model reproduces driver-deltaic variable relationships. Alternative correlation measures have been used to measure these relationships, yet TE does not have limitations in terms of capturing non-linearity or non-directionality. Another advantage in using TE is the ability to discern different delta dynamics through the process of validation; wind and discharge influence water level through different means, as a propagation of surface waves or flood waves, for example, and these drivers can be robustly compared for multiple locations and across time within the model. Tides, wind, and discharge all display different average timescales of information transfer and can have large ranges in those timescales. However, these properties may not be the same for all locations and at all time periods and with TE we can now track how well the model captures those spatio-temporal dynamics in the landscape of interest.

Validation in this study occurred through four means: comparison of couplings across space within a window, through time, for individual relationships over a range of time lags, and for the average timescales of information transfer. These means of validation offer bulk statistics that can be easily compared (number of couplings, average timescales of information transfer) between field and modeled data, or between different models. More complex validation measures (process networks to compare spatial relationships, plots of TE to look at the shape, strength, and number of significant lags for individual couplings) provide a more rigorous look at how the model propagates information over space and time. TE can also be used to validate different scales of information flow in model outputs; at WLD, TE occurs in the high frequency fluctuations of water level over ten-day windows. Comparison with modeled water level shows that these links are missing in the model at times, providing insight into model improvement. Deltaic systems are inherently dynamic and in this work TE links are captured over ten-day windows in correspondence with wind events, but this window length can be adjusted depending on the landscape of interest. Using TE for modeled data allows for the identification of the “event scale” of different forcings, such as multiple-month windows to track long-term changes in river discharge, or short daily windows to understand finer scale dynamics. There are also multiple scales within the signal that can be explored. As Sendrowski and Passalacqua [42] showed, information flow occurs for long term, daily, and high frequency water levels, and therefore model comparison could be explored for multiple scales for multiple time windows. Given that models can generate very long time series, TE can be used to track how well dynamics are captured within a window, as seen in the shape of TE plots, and across time, as seen in the number of couplings.

An issue of interest in modeling is assessing whether model simulations capture the dynamics of the system of interest. By comparing couplings of observed and modeled data, rather than only comparing time series at a location, we can assess the capability of the model to capture the specific behavior of the system. WLD is characterized by driver-water level couplings that occur at a certain timescale and strength. The agreement in the shapes, timescales, and strengths of the TE plots indicates that the dynamics of WLD are being captured by the model. This agreement is better for the channels than the islands, indicating an area where the model could be improved.

This study addresses the observed and modeled hydrodynamics at WLD. However, models can generate many other outputs including sediment concentration and bed elevation through time. Given that TE employs the probability density function of the data, it could be used as validation metric for multiple model outputs. In coastal systems, for example, sediment dynamics and hydrodynamics likely exhibit different relationships with external drivers; TE can be used to discern the differences in these relationships and provide insights into delta modeling.

Mutual information (MI) measures the amount of shared information or lagged synchronization between variables. Sendrowski and Passalacqua [42] demonstrated that WLD displays high MI among water levels and drivers as it is a small highly connected river delta. Daily and long-term water levels are strongly synchronized to tides, discharge, and wind, such that TE is not captured at these scales of water level much of the time [42]. MI was not fully explored as a validation tool in this work since it cannot measure directional couplings and provides less insight into the dynamics of forcings compared to TE. However, based on the analysis of Sendrowski and Passalacqua [42] and some results reported in this work, there is likely high MI from drivers to modeled water levels. Links not depicted in the modeled process networks are likely captured as strong MI relationships instead.

In this work we only focus on one source and one target node (discharge → water level at a location). However, it is possible that multiple drivers work to influence water levels together such as tides and wind acting synergistically to influence water levels. Recently, Goodwell and Kumar [76,77,78] proposed an approach to quantify multiple source-target couplings that could be used to identify the redundant and synergistic interaction of drivers on sinks.

## 5. Conclusions

Validation is an important step in the development of numerical models that are used to understand system dynamics and make forecasts of future landform change. However, many validation methods only focus on assessing spatial or temporal dynamics and they cannot measure couplings between different system variables. In this work we proposed using transfer entropy (TE) as a tool for hydrodynamic model validation. TE measures directional information transfer from one random variable to another and can quantify relationships among different random variables across space and through time. Using the Wax Lake Delta as a case study, we used TE to compare observed and modeled couplings of river discharge, tides, and wind with deltaic water levels on channels and islands. Validation was accomplished by comparing couplings within a ten-day window and across time. The model was able to reproduce relationships in terms of strength, timescale, and direction for channel and island water levels, and accurately tracked those dynamics through time, though with some differences. Differences in modeled and observed data were identified in the lack of high frequency water level fluctuations in the modeled water level, leading to underestimated couplings. The difficulty in modeling channel-island connectivity was also a source of model-field differences, resulting in the model reproducing channel couplings better than island couplings, prompting the need of models to include the spatial heterogeneity of vegetation in delta islands.

TE serves as an additional tool for model validation to other hydrodynamic and morphodynamic validation metrics in that it can quantify the couplings that characterize a system of interest, thus allowing assessment of whether the model captures its specific dynamics. 

## Figures and Tables

**Table 1:** Range and average timescale in hours of information transfer from external drivers and the high frequency fluctuations of observed island water levels (rows) to the high frequency fluctuations of observed island water levels (columns).

Field Data	Mike	Campground	Sherman	Greg	Pintail Island	Pintail Channel
Tide		(6–31.2), 22.4	(27–33), 29.9		29.0	(24.6–32.4), 26.4
Wind		(2.6–46.2), 17.5	(23.8–31), 27.4	5.0	(36–46), 41	(5.6–25.2), 18.3
Flow	(5.6–25.8), 18.9	(0.4–24.6), 12.1	(8–47.4), 26.1	(0.4–45.8), 31.1	(20.6–26.8), 24.1	(0.4–47.2), 20.6
Mike	(1.6–48.2), 21.9	0.4	(0.4–39.6), 6.5	(0.4–26.6), 6.8	(0.4–0.6), 0.40	(0.4–2.8), 0.76
Campground	(1.0–48.2), 11.3	(0.6–39.6), 4.7	(0.6–39.4), 5.6	(1–25.4), 6.6	(1.2–42.6), 4.9	(1.0–47.8), 7.4
Sherman	(1.6–23.8), 10.9	0.4	(1.4–48), 9.4	(1.6–26.2), 9.5	(0.4–2.4), 0.53	(0.4-47), 8.6
Greg	(0.4–32), 12.7	(0.4–0.6), 0.44	(0.4–8.8), 1.9	(2-45.6), 26	(0.4–0.6), 0.41	(0.4–48), 13.2
Pintail Island	(1.6–46.4), 20.9	(0.4–33), 5.4	(1.2–24.8), 10.9	(1.2–44.8), 15.2	(1.8–42.4), 10.7	(1.8-48), 17.6
Pintail Channel	(1.6–33), 17.2	(0.4–32.6), 1.6	(0.4–39.2), 8.7	(1.8–26.6), 9.6	(0.4–2.8), 0.47	(1.6–48), 17.4

**Table 2:** Range and average timescale in hours of information transfer from external drivers and modeled island water levels to modeled island water levels. The timescales for Sherman Island are grayed given that modeled Sherman water level is often out of the water.

Model Data	Mike	Campground	Sherman	Greg	Pintail Island	Pintail Channel
Tide			(6–46.5), 27.6			
Wind			(7.8–46.5), 33.6			
Flow			(11–44), 29.1			
Mike	(2.0–43.3), 15.7	0.5	(1.5–28), 17.4	(1.8–43), 12.1	(0.5–2.8), 1.2	(0.5–43.5), 9.9
Campground	(1.8–44.8), 29.9	(2.0–43.5), 18.6	(1.8–46.3), 19.3	(1.5-43.3), 15.5	(1.8–43.3), 27.7	(1.8–36.5), 19.1
Sherman	43	(42.5–45.5), 44	(0.8–3.3), 1.26	42.3	42.5	(42.8–43.3), 43
Greg	(0.5–1.8), 0.7	0.5	(1.5–46.3), 19.8	(1.5–43.3), 10.1	0.5	0.5
Pintail Island	(2–43.5), 15.9	0.5	(3.8–46.3), 18.4	(1.5–43),12.1	(43.3–46.3), 44.8	(2.0–43.3), 15.8
Pintail Channel	(2.0–2.3), 2.08	0.5	(0.8–27.8), 12	(2.0–43.3), 22.6	(2.5–43.5), 23	(2.0–43.3), 15.8

**Table 3:** Range and average timescale in hours of information flow from external drivers and observed channel water levels to observed channel water levels.

Field Data	Apex	Main	East
Tide	(21–39.8), 30.4	(30.3–32.3), 31.3	(18.2–30.8), 24.5
Wind	(38–44.7), 41.3	36.3	(14.3-35.5), 24.9
Flow	(41.7–47.2), 44.4	(34.8–42.5), 38.7	(15.5–33.2), 24.3
Apex	(2.0–2.3), 2.2	(0.3–2.3), 1.6	(0.3–1.7), 0.8
Main	(0.3–0.7), 0.4	(2.2–3.0), 2.6	0.3
East	0.3	(0.3–2.2), 1.3	(1.7–2.7), 2.1

**Table 4:** Range and average timescale in hours of information flow from external drivers and modeled channel water levels to modeled channel water levels.

Model Data	Apex	Main	East
Tide	46.5	(18–40), 29	(30.5-40.8), 35.6
Wind	41.5	(19.5–42), 30.8	(38-42.3), 40.1
Flow		(40–43.8), 41.9	(40–46.6), 43.1
Apex	44	(18.3-39), 28.6	(19–41.5), 30.3
Main		(18.8–39.8), 29.3	(19–39.5), 29.3
East		(20.5–38.5), 29.5	(34.8–35.3), 35

## Abbreviations

The following abbreviations are used in this manuscript:

IT	Information theory
MI	Mutual Information
TE	Transfer entropy
WLD	Wax Lake Delta
WLO	Wax Lake Outlet
